# Vibrio harveyi Exhibits the Growth Advantage in Stationary Phase Phenotype during Long-Term Incubation

**DOI:** 10.1128/spectrum.02144-21

**Published:** 2022-01-26

**Authors:** Calista Allen, Steven E. Finkel

**Affiliations:** a Molecular and Computational Biology Section, Department of Biological Sciences, University of Southern California, Los Angeles, California, United States; The Pennsylvania State University

**Keywords:** *Vibrio harveyi*, long-term survival, growth advantage in stationary phase, bacterial evolution, fitness advantage

## Abstract

The bioluminescent marine bacterium Vibrio harveyi can exist within a host, acting as a mutualist or a parasitic microbe, and as planktonic cells in open seawater. This study demonstrates the ability of V. harveyi populations to survive and adapt under nutrient stress conditions in the laboratory, starting in an initially rich medium. V. harveyi populations remain viable into long-term stationary phase, for at least 1 month, without the addition of nutrients. To determine whether these communities are dynamic, populations were sampled after 10, 20, and 30 days of incubation and examined for their competitive ability when cocultured with an unaged, parental population. While populations incubated for 10 or 20 days showed some fitness advantage over parental populations, only after 30 days of incubation did all populations examined outcompete parental populations in coculture, fully expressing the growth advantage in stationary phase (GASP) phenotype. The ability to express GASP, in the absence of additional nutrients after inoculation, verifies the dynamism of long-term stationary-phase V. harveyi populations, implies the ability to generate genetic diversity, and demonstrates the plasticity of the V. harveyi genome, allowing for rapid adaptation for survival in changing culture environments. Despite the dynamism, the adaptation to the changing culture environment occurs less rapidly than in Escherichia coli, possibly due to Vibrio harveyi’s lower mutation frequency.

**IMPORTANCE** Vibrio harveyi populations exist in many different niches within the ocean environment, as free-living cells, symbionts with particular squid and fish species, and parasites to other marine organisms. It is important to understand V. harveyi’s ability to survive and evolve within each of these niches. This study focuses on V. harveyi’s lifestyle outside the host environment, demonstrating this microbe’s ability to survive long-term culturing after inoculation in an initially rich medium and revealing increased competitive fitness correlated with incubation time when aged V. harveyi populations are cocultured with unaged, parental cultures. Thus, this study highlights the development of the growth advantage in stationary phase (GASP) phenotype in V. harveyi populations suggesting a dynamic population with fluctuating genotype frequencies throughout long-term, host-independent incubation.

## INTRODUCTION

Vibrio harveyi, a bioluminescent, ocean-dwelling microbe, occupies a number of different environments, with numerous ecological roles. V. harveyi can exist as planktonic cells in seawater, as a mutualistic microbe, or as a parasite. As a parasitic microbe, V. harveyi causes mass mortality in shrimp populations by disrupting stomach function upon attaching to the stomach’s chitinous lining (1, 2). Other marine organisms, such as pearl oysters, seahorses, and lobsters, are similarly plagued by V. harveyi colonization of the stomach lining (1). In mutualistic relationships, V. harveyi provides bacterial bioluminescence or specialized metabolic capabilities in order to occupy a nutrient-rich habitat within its host organism. Mutualistic relationships between V. harveyi populations and the hydrozoan Aglaophenia octadonta have been reported throughout areas of the Mediterranean Sea (3). It is proposed that A. octadonta provides nutrients for the *Vibrio* species within the chitinous structures forming the hydroid, and it is feasible that the bacteria may degrade the chitinous material into more usable forms for the host organism. Much of our knowledge on mutualistic relationships between bioluminescent bacteria and host organisms focuses on the relationship between marine *Vibrio* and squid. This relationship is established within the squid’s light organ where nutrients from the host are provided to the bacterium in exchange for the bacterium’s ability to produce bioluminescence for a variety of purposes, including intraspecific host communication, camouflage against moonlight, and attracting prey (1, 4–6).

While the specific establishment of V. harveyi symbionts within squid species is only recently characterized (1), in another *Vibrio* species, Vibrio fischeri, colonization is better understood. Euprymna scolopes, a squid species that is colonized solely by V. fischeri, hatches with no bacteria present in its two light organs, and bacterial cells in the surrounding seawater must overcome several physical and chemical obstacles and respond to other chemical cues to colonize the juvenile squids’ light organ crypts. This initial colonization by surrounding bacteria establishes a lifelong mutualism (7, 8). Once the bacteria successfully colonize the light organ, the bacteria divide and increase in density within the light organ, resulting in the induction of bioluminescence.

While V. harveyi exists within host environments, it is frequently found as planktonic cells in seawater in contrast to other closely related, bioluminescent bacterial species existing primarily within host organisms (9). Many studies confirm V. harveyi’s existence in the planktonic form, maintaining relatively high cell density, in both coastal and surface seawaters (4, 10). The existence of numerous possible niches for V. harveyi likely explains its ability to use a broad range of substrates for growth within laboratory environments (9–11). Much of the research on *Vibrio* species tends to focus on the bacterium’s role in relation to a host organism; the planktonic lifestyle of Vibrio harveyi remains less well understood. Further, Vibrio harveyi has not previously been examined for evidence of adaptive evolution throughout long-term incubation and is the focus of this study.

It is well established that Escherichia coli populations possess the ability to survive long-term batch culture for many years after inoculation into a rich medium such as LB without any further addition of nutrients (12–15). The inoculated culture initially experiences four phases (lag phase, exponential/logarithmic phase, stationary phase, and death phase) before transitioning into the final phase, long-term stationary phase (LTSP). During LTSP, subpopulations with beneficial mutations, including, for example, an enhanced ability to scavenge nutrients and/or improved ability to resist stress, increase in frequency, taking over the population (14–18). The benefit to cultures aging into long-term stationary phase was first observed by introducing a sample of a 10-day-old, aged E. coli population as a minority into a majority, 1-day-old parental population to examine the dynamics between the two populations in competition, revealing the enhanced relative fitness of cells from the aged population. When in competition with the parental population, the 10-day subpopulation overtakes the unaged cells within a few days, termed the growth advantage in stationary phase (GASP) phenotype; the aged population is ultimately able to drive the parental lineage to extinction (12, 14–16).

The GASP phenotypes observed within a competition experiment can be categorized into four different classes depending upon the final cell densities of the two populations (14). Class I populations distinctly outcompete the ancestral lineage at the conclusion of the experiment, typically exhibiting an at least 100-fold fitness advantage and frequently demonstrating the ability to drive the ancestral population to extinction. Class II GASP populations increase in frequency to achieve cell counts similar to that of the ancestral population but do not drive the parental population to extinction during the transition into long-term stationary phase; instead, both populations can coexist for long periods of time. Evolved populations that show an initial increase in cell density but do not succeed in surpassing or reaching cell densities equivalent to those of the ancestral population fall into class III. Finally, aged populations that never show any increase in frequency after introduction into the ancestral lineage and eventually die out do not possess the GASP phenotype and fall into class IV.

The ability to survive in multiple different environments would suggest that V. harveyi populations possess a level of genomic plasticity sufficient to provide enough genetic diversity to allow for adaptation and survival in these various niches. V. harveyi in planktonic form, living in the open water—where the availability of nutrients may remain low before encountering another host—suggests that the organism can survive under conditions akin to long-term stationary phase. After prolonged incubation in LTSP, populations may adapt to those conditions, potentially express the GASP phenotype after evolving under LTSP conditions, and thus could specialize more in an open seawater type environment without a host. V. fischeri, a bacterium occurring more strictly within a host, has been examined for the GASP phenotype and shows evidence for type II GASP with populations aged 7 (19), 10 (C. Maher, N. Bansal, and S. Finkel, unpublished results), or 22 days (20). While experimental design varied, in each experiment, cells achieved population densities similar to those of the unaged cells during coculture. V. harveyi, a free-living planktonic bacterium existing within multiple environments, could have more targets for adaptive mutations within its genome to manifest the GASP phenotype through long-term culturing in the laboratory. Potentially, costly mutualistic traits, such as bioluminescence which consumes ∼12% of Vibrio harveyi’s usable oxygen, may be lost or affected by long-term culturing, as selection for these traits may be relaxed within the free-living environment (21). This study aims to understand V. harveyi’s ability to survive into long-term stationary phase and to determine whether populations incubated for prolonged periods are able to adapt to LTSP conditions and manifest the GASP phenotype. The data presented demonstrate the expression of the GASP phenotype in aged V. harveyi populations, although the mode and tempo of acquiring the phenotype may take longer to manifest under laboratory conditions compared to those of Escherichia coli.

## RESULTS

### V. harveyi survives into long-term stationary phase when initially incubated in rich culture medium.

**FIG 1:**
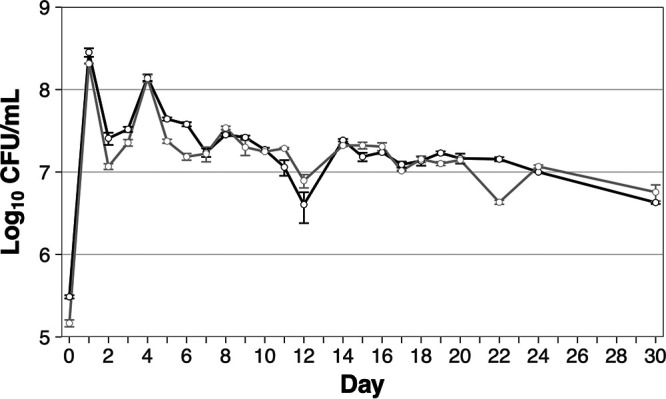
Vibrio harveyi long-term population dynamics in SWC media. Average viable cell counts (with standard error) throughout a month-long period of V. harveyi SFP118 (black) and nalidixic acid-resistant V. harveyi B-392 SFP119 (gray) monocultures aerated in test tubes at 30°C demonstrate the ability of V. harveyi cultures to persist into long-term stationary phase without an addition of nutrients (*n* = 3).

After a month-long batch monoculture incubation in the rich sea water complete medium (SWC), both the parental Vibrio harveyi and its nalidixic acid-resistant derivative (SFP119) experienced all five phases usually observed in the laboratory, with populations reaching cell densities of ∼2 × 10^8^ CFU/mL at the end of log phase (Fig. 1 and Table S1); both strains displayed a stationary phase lasting for 1 day. Similar to E. coli, following the peak stationary phase density, the population entered death phase where the cell density decreased 10-fold to ∼2 × 10^7^ CFU/mL. The cell density of the nalidixic acid-resistant strain decreased further during death phase compared to that of the parental population. After death phase, the cell densities for both strains increased, reaching concentrations of ∼10^8^ CFU/mL by day 4 of incubation, but only briefly before dropping again to ∼10^7^ CFU/mL by day 5, where the cell densities remained for the duration of the experiment. Upon continued incubation, the viable cell counts of both strains fluctuated with various “dips” in population densities, with both strains most frequently experiencing the dips simultaneously (Fig. 1, days 2 to 3, 10 to 12, 16 to 17). All replicates of the nalidixic acid-resistant strain experienced a dip on day 22, while the replicates of the unmarked strain did not. Despite these minor differences, the population densities of the two strains generally tracked closely with each other. The results shown in Fig. 1 highlight V. harveyi’s ability to survive into long-term stationary phase for at least 1 month without the addition of nutrients. Further, no significant differences in the long-term survivability between the parental and nalidixic acid-resistant strain were observed (Fig. 1), which is important for conducting competition experiments between the strains where at least one drug-resistant isogenic strain is necessary to differentiate strains for tracking the differently aged subpopulations during competition experiments. In E. coli GASP competitions, typically two antibiotic-resistant strains are utilized, allowing for separate tracking of each subpopulation; however, in V. harveyi, nalidixic acid was the only antibiotic tested which yielded a stable, spontaneously resistant strain despite examining a wide range of antibiotics (the strain is naturally resistant to streptomycin, spectinomycin, and ampicillin, and no spontaneous drug-resistant mutants were identified for kanamycin, chloramphenicol, or rifampin). V. harveyi’s ability to survive in long-period batch cultures under conditions of nutrient deprivation in LTSP led us to ask whether cells in aged cultures evolve to adapt to LTSP conditions and thus will exhibit a fitness advantage over the parental strain, akin to the GASP phenotype (12, 14, 16).

### V. harveyi exhibits the GASP phenotype.

**FIG 2:**
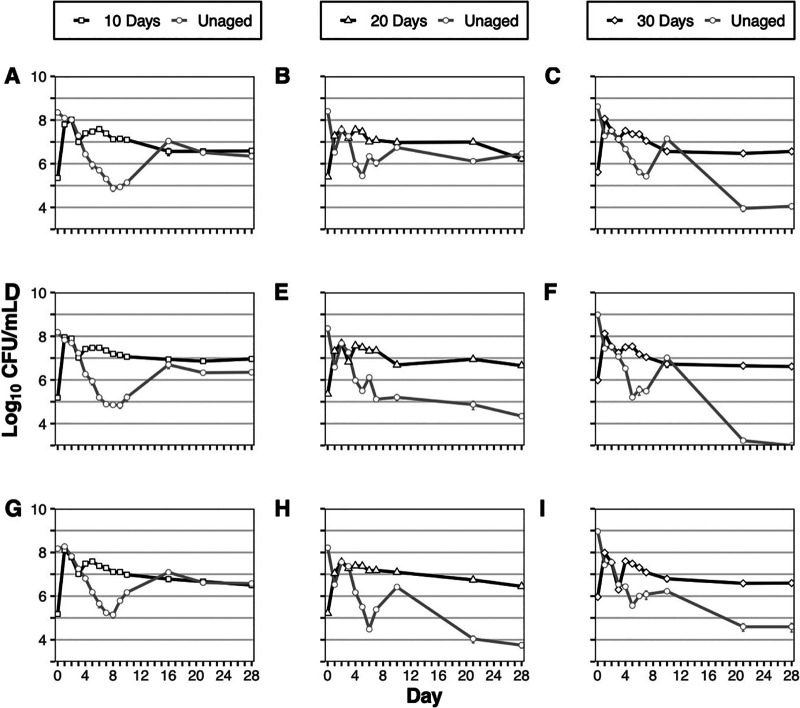
V. harveyi SFP118 populations aged in conditions for 10, 20, and 30 days competed against unaged V. harveyi SFP119. Each panel shows the average viable cell counts (with standard error, *n* = 3) for cocultures of previously aged cultures (black) for 10 days (A, D, and G, panels: squares), 20 days (B, E, and H, panels: triangles), or 30 days (C, F, and I, panels: diamonds) when introduced as a minority (5 μL) into a dense culture (5 mL) that has not been exposed to culture conditions (unaged in all panels, gray circles).

**FIG 3:**
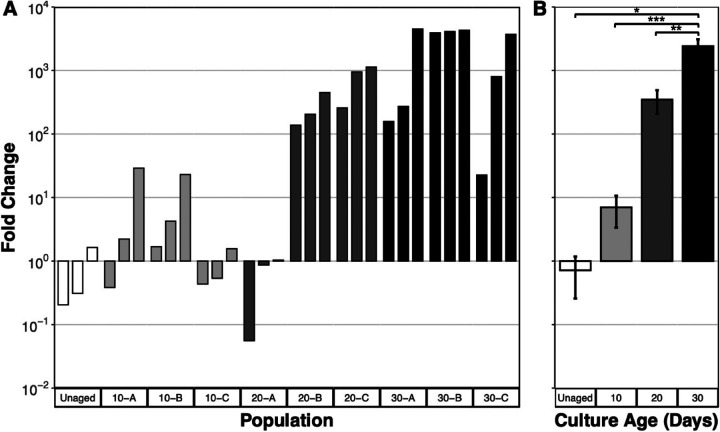
Competitive advantage of aged populations increases with previous exposure time to conditions. (A) Final ratios of the previously aged population to the unaged population at the conclusion (day 28) of each competition tested compared to the final ratio of competitions between two unaged populations (unaged). Each replicate was examined three times, and the replicates are grouped by having the same letter (A, B, or C, corresponding with Fig. 2) while the number corresponds to the incubation time prior to competition. (B) The final ratios for all competitions (*n* = 9) averaged (with standard error) by initial age with significant differences depicted from ANOVA (*, *P* < 0.05; **, *P* < 0.01; ***, *P* < 0.001).

To test for the expression of the GASP phenotype, the three cultures incubated into LTSP in Fig. 1 were sampled after 10, 20, and 30 days; samples were frozen at the appropriate time and utilized to initiate aged starter cultures for experiments (Fig. S1). A sample of each aged starter culture was used to inoculate three separate high-density, unaged, parental cultures to test for the expression of the GASP phenotype (Fig. 2). In total, competitive phenotypes were determined for nine replicates for each LTSP incubation time point (Fig. 3A). In addition to the coculture competitions between aged population samples and the unaged strain, coculture competitions between two unaged populations, one strain possessing nalidixic acid resistance and one strain unmarked, were performed by inoculating a small sample (5 μL) of each unaged population into one test tube to serve as an unaged control (Fig. 3A, unaged); the unaged cocultures reached similar population densities for each population at the conclusion of the competition experiment (Fig. 3B, unaged), revealing no consistent advantage for either unaged parental strain. The unaged competitions provide a baseline for comparison of the results from cocultures with aged subpopulations. The competitions performed between the aged populations and unaged V. harveyi revealed that populations incubated into LTSP can express the GASP phenotype. The relative competitive fitness of the aged populations compared to that of the parental population increased with incubation time in LTSP, with the GASP phenotype shifting from class II after 10 days of prior incubation to class I after 20 to 30 days (Fig. 2 and 3).

Each panel in Fig. 2 represents average population densities of competitions performed, in triplicate, from each of the three originally aged samples against the unaged parent. Ten-day V. harveyi populations (Fig. 2A, D, and G) in competition with the unaged parent (Fig. 2) grew in the presence of the parental cells throughout the early time points of the experiment, outnumbering parental cells by ∼100-fold by day 8 of coincubation; however, with continued incubation (between days 10 and 16), the parental population increased in population density, ultimately reaching densities similar to those of the “evolved” population. This result can be classified as a class II GASP phenotype (14).

Populations sampled further into long-term stationary-phase incubation at 20 days (Fig. 2B, E, and H), allowing additional time for cells to evolve under LTSP conditions, showed a more robust GASP phenotype. Similar to the populations aged for 10 days, the 20-day populations exhibited higher relative fitness than the unaged population throughout early long-term stationary phase, but here, the 20-day populations outnumbered the parental majority by ∼100-fold by day 5 rather than on day 8 as observed for 10-day-old populations. As coculture incubation continued, the phenotypes observed for each of the 20-day aged samples started to diverge. One 20-day sample (Fig. 2B and Fig. 3, 20-B) ultimately converged with the parental population by the end of the experiment, similar to what is observed with 10-day populations. However, the two other samples of 20-day populations clearly outcompeted the unaged population by more than 100-fold by the end of the experiment (Fig. 2E and H and Fig. 3, 20-A and 20-C).

The populations incubated for 30 days prior to competition showed the strongest GASP phenotypes (Fig. 2C, F, and I). Unlike cultures aged for 10 or 20 days, cells from the 30-day-old populations first showed enhanced relative fitness on day 1, and the enhanced fitness continued into early long-term stationary phase, where replicates of all three aged population samples examined outnumbered the parental population. However, the parental populations rebounded briefly: ultimately, the 30-day populations dominated the culture by the end of the experiment. Thirty-day populations possessed an ∼100-fold relative fitness advantage for two of the three examined samples (Fig. 2C and I), with the third sample showing an even stronger phenotype, consistently driving the parental population below the limit of detection by the conclusion of the experiment (Fig. 2F).

### The V. harveyi GASP phenotype strengthens over time.

Our results show that the strength of the GASP phenotype is correlated with the length of long-term stationary phase incubation time. To provide a summary of each competition and to determine whether culture age correlates with competitive ability, the ratio of the unmarked aged population to the nalidixic acid-resistant unaged population at the conclusion of each coculture experiment was calculated and is plotted in Fig. 3A (and Table S2). Since three independent cultures were originally aged and each of these was examined for the GASP phenotype in triplicate, a total of nine replicates are shown for each time point. In addition to the GASP competitions, the ending ratios of the coculture competitions between the two unaged populations are included in this analysis as a baseline for relative fitness between the strains (Fig. 3A, unaged). Figure 3A and Table S2 show the ratios of the final population yields at the end of each individual competition performed. Ten-day populations sampled from replicates 10-A and 10-B outnumbered the parental populations by approximately 10-fold, while samples from replicate 10-C in competition reached cell densities similar to those of the parent. Samples from replicate 20-A had relative competitive fitness lower than and reached population densities similar to those of the parental population, while populations 20-B and 20-C successfully outcompeted the parental population. Each of the replicates aged to 30 days outcompeted the parental populations by at least 1,000-fold.

The relative fitness ratios were then averaged for each incubation time (unaged, 10 days, 20 days, and 30 days) prior to coculture competition to determine the correlation between relative competitive fitness and incubation time in LTSP. On average, cells aged for 10 days could express the GASP phenotype and outnumber unaged cells by at least 7-fold. When aged for 20 days, aged cells overtook the population, outnumbering the unaged cells by ∼350-fold on average. Cells aged for 30 days exhibited the strongest GASP phenotype and had final cell ratios that are significantly different from those of all other competitions (analysis of variance [ANOVA], *P* = 1.92 × 10^−2^ for unaged, 7.46 × 10^−4^ for 10-day, and 3.76 × 10^−3^ for 20-day). On average, the 30-day populations outcompeted the unaged cells by ∼2,400-fold at the conclusion of the coculture competition.

## DISCUSSION

This study examines the long-term survivability of Vibrio harveyi and whether the GASP phenotype is manifested in this bacterial species as it is in Vibrio fischeri and Escherichia coli. By inoculating V. harveyi into a rich medium and assessing the viable population density over time, it was determined that V. harveyi maintains viability throughout long-term stationary-phase culture for at least 30 days (Fig. 1). Previously, it has been shown that V. harveyi populations maintain viable counts up to 14 days with a slight reduction in viability, but this was observed in a larger volume of marine broth, a rich medium similar to the medium used in this study (22). Furthermore, our study provides evidence that V. harveyi cultures acquire the GASP phenotype throughout LTSP incubation, adding another bacterial organism to the pantheon of GASP. The correlation between the relative strengthening of the GASP phenotype with LTSP incubation time, and the similarity of GASP phenotypes between replicate aged cultures (Fig. 2 and 3), provides evidence for cell turnover and altered genotype frequencies throughout long-term incubation, suggesting that adaptive evolution is occurring. Similar to E. coli, the aging V. harveyi parental population is likely being replaced with subpopulations selected for their enhanced LTSP survival by possessing beneficial mutations, allowing for GASP phenotype progression (14, 15).

Though aged V. harveyi populations manifest the GASP phenotype, there are some notable differences from the phenomenon originally observed in E. coli. While V. harveyi cells aged for 10 days can initially overtake unaged populations, this did not occur in all replicates (Fig. 3A). This is unlike populations of E. coli aged for 10 days in rich medium, where virtually all aged populations drive the unaged, parental population below the limit of detection (14), possibly due to differences in mutation frequency and population yield after exponential phase, as discussed below. Further, when cells from 10-day-old V. harveyi cultures did initially overtake the majority population, the aged and unaged populations were at equal cell densities by the end of the experiment (Fig. 3A and B), exhibiting a class II GASP phenotype (14). This is consistent with previous studies that found that the closely related organism, V. fischeri, displays a class II GASP phenotype after aging for 7 days or 22 days in LTSP (19, 20).

When using the final cell ratio of the aged to unaged populations in the coculture competitions as a measure of relative fitness, a significant strengthening of the GASP phenotype was observed with the length of LTSP incubation time prior to competition. When the populations were aged for 20 days, two of the three aged populations examined for GASP demonstrated a class I phenotype (Fig. 2E and H), compared to the 10-day samples which all exhibited a class II GASP phenotype (Fig. 2D and G); at 30 days, all populations exhibited a class I GASP phenotype (Fig. 2C, F, and I). A majority of the competitions between 30-day populations and unaged populations resulted in the detection of only the aged populations at the conclusion of the experiment, with the unaged population frequently below the limit of detection, highlighting the increase in relative fitness throughout LTSP incubation (Fig. 3).

As a population ages, the observed strengthening of the GASP phenotype is likely due to an accumulation or replacement of novel mutations providing enhanced survival in LTSP. This replacement of mutations and accumulation throughout time have been confirmed for E. coli, where populations aged for 10 days possess numerous genotypes. In one experiment, sequencing 10-day-old clones from 4 different aging populations revealed 27 novel mutations with no detection of the parental genotype (15). When examining the final subpopulation ratios in V. harveyi competitions, it is clear that 30-day populations exhibit the strongest GASP phenotype; however, it is worth examining the coculture dynamics over the course of all competitions. While V. harveyi populations aged for only 10 days did not exhibit a strong GASP phenotype relative to 20- and 30-day populations, the 10-day populations were the most competitive against the parent at day 10 of coincubation, showing the highest relative cell density for that time point (Fig. 2A, D, and G compared to all other panels on day 10). After 10 days of aging in monoculture, cells likely possessed mutations that allow for better survival through day 10 compared to the parent, but the benefit received from the mutations at day 10 did not continue further into LTSP or under conditions of coincubation, thus resulting in a weaker GASP phenotype at the conclusion of the experiment. Further aging in monoculture prior to competition is necessary for long-term competitiveness against the parent in prolonged coculture experiments. However, despite the stronger GASP phenotype, 20- or 30-day samples showed reduced fitness in coculture on day 10, suggesting a form of antagonistic pleiotropy; mutations beneficial in extended LTSP may not be beneficial at earlier time points and are thus not selected for early on. The data support a model in which the genetic composition of the aging population is highly dynamic and is changing throughout LTSP incubation. These genetic differences may cause the GASP phenotype to shift from class II at 10 days of aging to class I upon further incubation where the aged population drives the unaged population below the limit of detection, similar to the phenomenon observed in E. coli at 10 days of aging. The different competitive phenotypes and the overall strengthening of the GASP phenotype correlated with culture age provide evidence for dynamic V. harveyi populations during LTSP.

While each of the aged populations achieved a strong GASP phenotype, likely due to beneficial mutations for enhanced LTSP survival, the evolutionary paths within the aged populations almost assuredly differ from each other and differ from what is observed for E. coli. Population A (Fig. 2A to C and Fig. 3A, 10-A, 20-A, and 30-A) did not acquire the class I GASP phenotype until aged for 30 days, compared to populations B and C, which started to exhibit a stronger GASP phenotype by day 20. It is probable that each population accrued different mutations, some of which provide a stronger advantage during LTSP. This provides evidence for multiple paths of evolution all ultimately resulting in improved relative fitness during LTSP. The dynamics of nutrient availability within the aging cultures throughout LTSP incubation are unknown, and may differ significantly as cultures progress from 10 to 30 days. Further, changes in nutrient availability will likely differ from culture to culture depending on what genotypes are present within a given population. As novel mutations appear, the shift in nutrient availability, and thus selection for different mutations within each culture, likely creates the differences observed in the GASP phenotypes, yet all mutants improve fitness over time. Both the environments and the specific paths of evolution are complex, and the “beneficial” mutations for each aging *Vibrio* population may differ from each other and from those observed in E. coli populations.

Why the GASP phenotype is not fully manifested by day 10 in *Vibrio* populations, both as observed here and in the studies of V. fischeri (19, 20), compared to that in 10-day E. coli populations, is not understood; however, we can speculate. One important difference between the population dynamics of E. coli evolving within LB cultures and V. harveyi evolving within SWC cultures is the maximum population density achieved by each species in stationary phase. E. coli populations reach a maximum population density (14) at least one order of magnitude greater than that observed in V. harveyi (∼5 × 10^9^ compared to ∼2 × 10^8^ CFU/mL). During exponential-phase growth, mutations are introduced within the population primarily through random errors made during DNA replication. Each of these mutations can be beneficial, neutral, or deleterious to the individual bacterium. The greater numbers of cells in E. coli cultures almost assuredly leads to additional genetic diversity within the larger population. Therefore, E. coli populations have a greater chance of possessing strong beneficial mutations. After death phase, similar population densities are observed for E. coli and V. harveyi populations, with both species entering into long-term stationary phase at ∼2 × 10^7^ CFU/mL. After death phase, cells possessing beneficial mutations for LTSP survival increase in frequency within the population. E. coli, generating enough genetic diversity during exponential phase, possesses genotypes with a strong GASP phenotype by day 10, while V. harveyi’s lower cell yield throughout exponential phase reduces the chance for a strong beneficial mutant present early on and, thus, necessitates further incubation with cell turnover for a class I GASP population to appear.

This model is further supported when examining the differences in spontaneous mutation frequency between E. coli and *Vibrio* species. Previous studies report that the *Vibrio* genus has a base-substitution mutation rate lower than that of other bacteria. V. fischeri has a rate of 2.07 × 10^−10^ mutations/base pair/generation and Vibrio cholerae has a rate of 1.07 × 10^−10^ (23). Per genome, this means that V. fischeri experiences 8.85 × 10^−4^ base-pair substitutions and V. cholerae experiences 4.38 × 10^−4^ base-pair substitutions when factoring in genome sizes (∼4.23 × 10^6^ and ∼4.09 × 10^6^, respectively). Estimates for E. coli show a mutation rate of 2.2 × 10^−10^ per nucleotide, but when factoring in genome size (∼4.6 × 10^6^ bp), this results in 1 × 10^−3^ mutations per genome, much greater than the frequency observed for *Vibrio* strains (24). The generation of fewer mutations in *Vibrio*, along with the decreased cell yield at stationary phase, may contribute to the delay in the manifestation of a strong GASP phenotype within the *Vibrio* genus simply because the chance of getting a beneficial mutation within the *Vibrio* population is lower than that within E. coli. However, after more time to generate (by chance) and accumulate beneficial mutations during the dynamic LTSP, a strong GASP phenotype can occur in V. harveyi. While the specific mutations present within the aged populations of V. harveyi and the mutational spectrum of V. harveyi populations throughout time will need to be determined, this study has revealed the expression of the GASP phenotype in Vibrio harveyi and the shift in phenotype strength through long-term stationary-phase culturing, reflecting a level of genotypic adaptation not observed previously.

## MATERIALS AND METHODS

### Bacterial strains and growth medium.

SWC, a rich culture medium, was utilized for both liquid culturing and plating of V. harveyi. SWC is an artificial seawater medium containing sodium chloride (2.33%), magnesium sulfate (1.85%), calcium chloride (0.22%), and potassium chloride (0.11%), with peptone (0.50%), glycerol (0.30%), and yeast extract (0.05%) as carbon sources (25–27). V. harveyi strain SFP118, derived from B392 (MAV/Photobacterium fischeri/Beneckea harveyi) (28, 29), and a spontaneous nalidixic acid-resistant mutant (SFP119) were utilized for this study. SFP119, the spontaneous nalidixic acid-resistant strain, was isolated from SFP118 using a previously published protocol (19, 30, 31). Briefly, antibiotic-sensitive cells of SFP118 were plated onto SWC plates containing 20 μg/mL nalidixic acid to select for spontaneously resistant mutants able to form colonies in the presence of the antibiotic (16). The survival dynamics of populations initiated from these resistant colonies were screened throughout LTSP to identify a mutant strain phenotypically identical to the parental strain in the absence of the drug selection to be utilized for tracking throughout coculture incubation between aged and unaged strains. (Other antibiotics, streptomycin, spectinomycin, ampicillin, kanamycin, chloramphenicol, and rifampin, were tested at various concentrations in a manner similar to that of nalidixic acid, but we were unable to select for a reliable spontaneously resistant mutant population.)

### Initiation of cultures.

For each experiment, cultures were inoculated from a frozen stock (stored in SWC plus 20% glycerol at −80°C) into 18 by 150 mm borosilicate test tubes containing 5 mL of SWC medium. Cultures were incubated with aeration for 2 days at 30°C in a rolling drum (TC-7; New Brunswick Scientific, Edison, NJ) to achieve maximal population density. These starter cultures were used to initiate experiments by inoculating fresh medium at a 1:1,000 (vol/vol) dilution. At least three biological replicates were generated in this manner and used for all experimental conditions. For the experiments depicted in Fig. 1, this transfer represents day 0. After 10 days, 20 days, and 30 days of incubation, 100 μL of each of the biological replicates was sampled and frozen in SWC plus 20% glycerol at –80°C for utilization in GASP experiments (Fig. S1).

### GASP competition experiments.

For the GASP competitions, “aged” starter cultures were initiated from the frozen populations described above (three for each age) and incubated for 2 days to achieve high cell density. After reaching high cell density, a sample of each aged starter culture was used to inoculate three separate high-density, unaged, SFP119 parental cultures possessing resistance to nalidixic acid at a 1:1,000 (vol/vol) ratio to establish day 0 of the GASP competition (Fig. S1). Each of the unaged, parental cultures inoculated in the GASP competitions was started from the same original parental starter culture in the manner described above. Each of the unaged, parental cultures was incubated for 2 days, prior to initiation of the GASP experiment, to achieve high cell density. This positioned the parental culture at high population density and cells from the aged culture at low population density at the start of the GASP competition. Each aged starter culture was examined in triplicate, thus generating nine total replicate GASP competitions for each age. After GASP competitions were initiated, cell densities of the two populations throughout the coincubation period were measured over 28 days (Fig. 2). To serve as a control for comparison to the aged GASP competitions, three replicate coculture competitions between two unaged strains (an unaged, unmarked population and an unaged, nalidixic acid-resistant population) were performed with both strains inoculated at similar densities. This set each population at a similar low cell density at the start of the competition.

### Monitoring survival dynamics.

Population densities for the monoculture, control, and GASP competitions were determined by quantifying viable cell counts by determining the titers of appropriate dilutions of cells and plating in the presence or absence of nalidixic acid as appropriate. The limit of detection for this method is <1,000 CFU/mL (32). For monoculture experiments, dilutions were plated on SWC agar to determine viable cell counts of the total population, in this case, the one strain present in the culture. For cocultures, total cell counts, including both the unaged and aged populations, were determined by plating on SWC, while the unaged population (nalidixic acid-resistant strain) density was determined independently by plating onto SWC plates containing 20 μg/mL nalidixic acid. The aged population density was then calculated by subtracting the cell counts observed on the nalidixic acid-containing SWC plates from the total observed counts on the SWC plates. This method was similarly applied for control competitions between the two parental strains (SFP118 and SFP119). At day 0 in GASP experiments, the unmarked, aged population was undetected because it comprised so little of the total population that viable counts from the nalidixic acid-resistant strain and total counts were similar. In this case, the aged population was assigned a value 1,000-fold lower than that of the detected nalidixic acid-resistant strain since it was inoculated at a 1:1,000 (vol/vol) ratio. Due to the limit of detection for the aged strain, after day 0, if the aged population was undetected at a time point but was at or above the unaged population counts in the previous or subsequent time point, it was assigned counts equal to those of the unaged, nalidixic acid-resistant strain. This correction was necessary for only one or two time points within the first 4 days of coculture incubation (Fig. 2, ∼day 1 to 4, Fig. 3A, day 2, Fig. 3B, day 2, Fig. 3C, day 2 to 4, Fig. 3E, day 2, Fig. 3F, day 2 to 3, Fig. 3G, day 1, Fig. 3H, day 2 to 3, Fig. 3I, day 2). For each experiment, viable cell counts were averaged between biological replicates for each strain/population with the standard error indicated (Fig. 1 and 2). Standard error was calculated using the standard deviation in R of the viable cell counts divided by the square root of the length of the data.
