# Supplementary material for: Vibrio harveyi Exhibits the Growth Advantage in Stationary Phase Phenotype during Long-Term Incubation
Source: Microbiol Spectr. 2022 Jan 26;10(1):e02144-21. doi: 10.1128/spectrum.02144-21 (PMC8791185; doi:10.1128/spectrum.02144-21)
Supplement: SUPPLEMENTAL FILE 1 — Supplemental material. Download SPECTRUM02144-21_Supp_1_seq7.pdf, PDF file, 0.2 MB [file spectrum02144-21_supp_1_seq7.pdf]

Supplemental Table 1. Average CFU/ml and standard error of monoculture *Vibrio harveyi* survival curves (Fig. 1)

	Parental		Nalidixic Acid Resistant	
**Day**	**CFU**	**Standard Error**	**CFU**	**Standard Error**
**1**	2.8×10^{8}	3.3×10^{7}	2.1×10^{8}	1.8×10^{6}
**2**	2.6×10^{7}	4.4×10^{6}	1.2×10^{7}	9.1×10^{5}
**3**	3.3×10^{7}	2.4×10^{6}	2.3×10^{7}	2.0×10^{6}
**4**	1.4×10^{8}	1.3×10^{7}	1.4×10^{8}	1.5×10^{7}
**5**	4.4×10^{7}	1.9×10^{6}	2.4×10^{7}	1.3×10^{6}
**6**	3.8×10^{7}	2.2×10^{6}	1.5×10^{7}	1.4×10^{6}
**7**	1.7×10^{7}	2.1×10^{6}	1.7×10^{7}	3.4×10^{6}
**8**	2.8×10^{7}	1.2×10^{6}	3.4×10^{7}	1.6×10^{6}
**9**	2.6×10^{7}	1.3×10^{6}	2.0×10^{7}	4.1×10^{6}
**10**	1.9×10^{7}	9.7×10^{5}	1.8×10^{7}	1.8×10^{5}
**11**	1.1×10^{7}	2.5×10^{6}	1.9×10^{7}	4.8×10^{5}
**12**	4.0×10^{6}	1.6×10^{6}	7.9×10^{6}	1.5×10^{6}
**14**	2.4×10^{7}	8.0×10^{5}	2.1×10^{7}	6.3×10^{5}
**15**	1.5×10^{7}	1.8×10^{6}	2.1×10^{7}	1.9×10^{6}
**16**	1.7×10^{7}	7.3×10^{5}	2.0×10^{7}	2.3×10^{6}
**17**	1.2×10^{7}	1.0×10^{6}	1.0×10^{7}	1.8×10^{5}
**18**	1.4×10^{7}	1.7×10^{6}	1.4×10^{7}	1.3×10^{6}
**19**	1.7×10^{7}	8.4×10^{5}	1.3×10^{7}	6.6×10^{5}
**20**	1.5×10^{7}	2.0×10^{6}	1.4×10^{7}	1.1×10^{6}
**22**	1.4×10^{7}	8.0×10^{5}	4.3×10^{6}	2.4×10^{5}
**24**	1.0×10^{7}	0.0×10^{0}	1.2×10^{7}	4.8×10^{5}
**30**	4.3×10^{6}	1.6×10^{5}	5.7×10^{6}	1.3×10^{6}

Supplemental Table 2. Ratios of aged CFU/mL to unaged CFU/mL and standard error at the conclusion of a GASP assay

	Unaged Control	10 Day	20 Day	30 Day
**All Populations**	0.72 ± 0.46	7.01 ± 3.65	347.95 ± 140.52	2416.83 ± 673.08
**Population A**		10.54 ± 9.25	0.65 ± 0.30	1643.27 ± 1428.74
**Population B**		9.64 ± 6.72	263.63 ± 94.63	4100.00 ± 115.47
**Population C**		0.84 ± 0.36	779.56 ± 266.42	1507.21 ± 1119.07

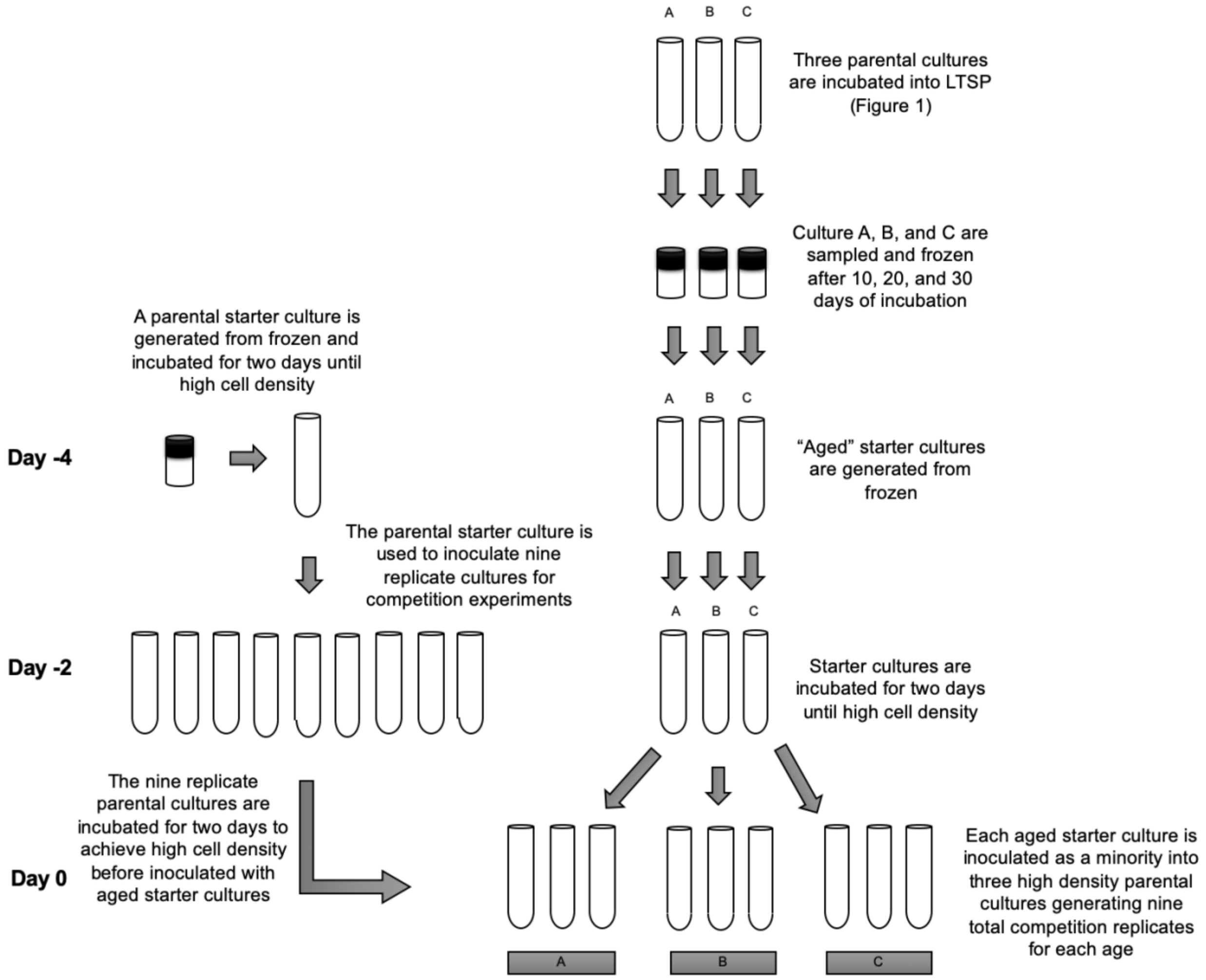

Supplemental Figure 1. Experimental Design for GASP Competition Assays. Three parental cultures are incubated into long-term stationary phase and are sampled after 10, 20, and 30 days. The frozen samples are used to generate starter cultures and incubated to high cell density. A sample of the aged starter cultures is inoculated as a minority into three replicate parental cultures that have been incubated for 2 days to achieve high cell density. Each of the three aged cultures are tested in triplicate generating nine total replicates for each age.
